# Distress screening in patients with high-grade glioma: diagnostic accuracy in relation to a structured clinical interview in a multicenter cluster-randomized controlled trial

**DOI:** 10.1007/s00520-025-09810-1

**Published:** 2025-07-31

**Authors:** Robert Kuchen, Susanne Singer, Melanie Schranz, Lorenz Doerner, David Rieger, Joachim P. Steinbach, Michael W. Ronellenfitsch, Martin Voss, Almuth F. Kessler, Vera Nickl, Martin Misch, Julia Sophie Onken, Marion Rapp, Minou Nadji-Ohl, Marcus Mehlitz, Jürgen Meixensberger, Michael Karl Fehrenbach, Naureen Keric, Florian Ringel, Jan Coburger, Carolin Weiß Lucas, Jens Wehinger, Friederike Schmidt-Graf, Marcos Tatagiba, Ghazaleh Tabatabai, Melina Hippler, Mirjam Renovanz

**Affiliations:** 1 Institute of Medical Biostatistics, Epidemiology, and Informatics (IMBEI), University Medical Center Mainz, Mainz, Germany; 2 Department of Neurology & Interdisciplinary Neuro-Oncology, University Hospital Tübingen, Hertie Institute for Clinical Brain Research, Eberhard Karls University, Tübingen, Germany; 3 Center for Neuro-Oncology, Comprehensive Cancer Center Tübingen-Stuttgart, University Hospital Tübingen, Tübingen, Germany; 4 Department of Neurosurgery, University Hospital Tübingen, Tübingen, Germany; 5 Dr. Senckenberg Institute of Neurooncology, Goethe University, Frankfurt, University Cancer Center Frankfurt (UCT), Frankfurt Cancer Institute (FCI), and German Cancer Consortium (DKTK), Partner Site Frankfurt, Frankfurt Am Main, Germany; 6 Department of Neurosurgery, University Hospital Würzburg, Würzburg, Germany; 7 Department of Neurosurgery, Charité - University Medical Center, Berlin, Germany; 8 Department of Neurosurgery, University Hospital Düsseldorf, Düsseldorf, Germany; 9 Department of Neurosurgery, Klinikum Stuttgart, Katharinen Hospital, Stuttgart, Germany; 10 Department of Neurosurgery, Klinikum Der Barmherzigen Brüder, Trier, Germany; 11 Department of Neurosurgery, University Hospital Leipzig, Leipzig, Germany; 12 Department of Neurosurgery, University Medical Center Mainz, Mainz, Germany; 13 Department of Neurosurgery, University Hospital Ulm, Ulm, Germany; 14 Center for Neurosurgery, University Hospital Cologne, Cologne, Germany; 15 Department of Neurology, Klinikum Ludwigsburg, Ludwigsburg, Germany; 16 Department of Neurology, Klinikum Rechts Der Isar, Technical University Munich, Munich , Germany; 17 University Center for Tumor Diseases (UCT), University Medical Center Mainz, Mainz, Germany; 18 Comprehensive Cancer Center Mecklenburg-Vorpommern (CCC-MV), Department of Quality of Life in Oncology University Medical Centre Rostock, Rostock, Germany

**Keywords:** High-grade glioma, Structured clinical interview (SCID), Psychological burden, Screening tool, Mental disorder, Emotional distress

## Abstract

**Purpose:**

Structured clinical interviews, such as the Structured Clinical Interview for DSM (SCID), are considered the gold standard for diagnosing mental disorders but are challenging in routine clinical use due to their length. Therefore, screening instruments to identify the need for further assessment are required. The National Comprehensive Cancer Network Distress Thermometer (DT) screens for psychological distress, while the Emotional Functioning (EF) scale of the European Organisation for Research and Treatment of Cancer Quality of Life Questionnaire Core (EORTC QLQ-C30) assesses emotional functioning. Both are frequently used in clinical routine. Additionally, three brief screening questions (TSQ), specifically developed for patients with glioma and integrated into doctor–patient consultations, may also be used for screening. This study aimed to evaluate the ability of the three tools to identify patients with psychiatric comorbidities as diagnosed by the SCID.

**Methods:**

Using data from glioma patients treated at 13 German hospitals participating in a cluster-randomized trial, discriminative abilities were assessed using receiver operating characteristic (ROC) curves and corresponding areas under the curve (AUCs). Confidence intervals (CIs) were estimated, and hypothesis tests were conducted using bootstrapping.

**Results:**

Of the 691 patients interviewed, 31% presented with at least one mental disorder. The EF scale demonstrated the best discriminative ability (AUC 0.70, 95% CI: 0.66–0.74), followed by the DT (AUC 0.69, 95% CI: 0.62–0.76), and the TSQ total score (AUC 0.61, 95% CI: 0.55–0.66).

**Conclusion:**

While all three tools performed better than random chance, none demonstrated convincing discriminative ability in identifying psychiatric comorbidities. In practice, screening tools can identify a substantial proportion of patients with mental disorders, however at the cost of a considerable number of false negatives.

**Supplementary Information:**

The online version contains supplementary material available at 10.1007/s00520-025-09810-1.

## Introduction

The diagnosis of a high-grade gliomas is often associated with elevated emotional distress [1, 2] Emotional distress is defined as a subjective experience of psychological discomfort that may not meet the diagnostic criteria for a mental disorder but can still affect well-being and indicate a need for specialized services. Nearly one in two glioma patients experience a clinically relevant level of emotional distress [3–5] and may benefit from psycho-oncological care [3].

Similarly, many glioma patients exhibit reduced levels of emotional functioning. Emotional functioning refers to an individual’s ability to express and cope with emotions and encompasses overall emotional well-being. In this sense, it can be viewed as the opposite of distress.

Evaluating emotional distress or functioning in glioma patients is challenging due to neurocognitive deficits that may hinder their ability to complete conventional questionnaires [4]. An alternative approach may therefore be adopted, in which three screening questions (TSQ), specifically developed for patients with glioma [5], are asked by the health care professional during the consultation.

Comorbid mental disorders are closely related to both emotional distress and emotional functioning [6, 7]. However, unlike emotional distress and emotional functioning, mental disorders are conditions meeting established clinical criteria. While the former are measured dimensionally, the latter are assessed categorically. In many countries, reimbursement of mental health care requires a diagnosis. [8, 9]. To diagnose mental disorders, an in-depth examination is required. Structured clinical interviews, such as the Structured Clinical Interview for the Diagnostic and Statistical Manual of Mental Disorders (SCID) [10], are considered the gold standard for this. However, due to their length, such interviews are impractical for routine use, and quick screening tools are often used to identify individuals likely to have mental disorders before further diagnostic procedures are conducted.

The aim of this study was to compare the ability of several screening tools—the TSQ developed for patients with glioma [5], the Distress Thermometer (DT) [11, 12], and the Emotional Functioning (EF) scale of the EORTC QLQ-C30 [13] — to identify the presence of at least one comorbid mental disorder as diagnosed by the SCID, thereby supporting the efficient use of clinical resources in accordance with standard practice [14]. Furthermore, we investigated whether the TSQ were more predictive as an aggregated sum or as individual questions.

Previous studies examining the association between the DT and the SCID, reporting area-under-the-curve (AUC) values of approximately 0.80 [15, 16]. In patients with laryngeal cancer, the EF also demonstrated good discriminative power for identifying mental disorders (AUC: 0.8) [17], supporting its potential as a screening tool. In contrast, the TSQ have not yet been compared with other questionnaires or clinical interviews.

## Methods

### Study design

All data were collected as part of the study *Glioma patients in outpatient care-optimization of psychosocial care in neuro-oncological patients* [18] (GLIOPT), a 2019–2023 cluster-randomized trial in Germany involving high-grade glioma patients. The main research question of the GLIOPT study was whether screening patients during patient-doctor consultation via the TSQ [5] could help identify those in need of specialized psychosocial care.

Of the 13 participating hospitals, 6 were randomized to the intervention group (IG), in which physicians used the TSQ during patient consultations. In the other 7 hospitals (control group, CG) the DT was instead handed out (via self-report) [11, 12]. Patients were assessed at three points: t1, t2, and t3. Time points t1 and t2 occurred on the same day (t1: before the doctor consultation, t2: shortly after the consultation), while t3 was scheduled three months later. For our purposes, only t1 and t2 are relevant.

At t1, demographic and clinical data were collected, and the EORTC QLQ-C30 [19] was administered. In the CG, the DT was also administered at t1. In the IG, the TSQ were asked during doctor consultation.

At t2, additional socio-demographic data were collected and the SCID [10] was administered by a trained study nurse. For information on inclusion criteria and patient enrollment see, Appendix A1.

### Instruments

#### The structured clinical interview

The SCID [10] is a structured interview to identify psychiatric disorders based on the criteria of the Diagnostic and Statistical Manual of Mental Disorders (DSM). We used its clinical version (5) designed for the diagnosis of conditions, such as mood, anxiety, psychotic, and substance use disorders. The following parts were used: Major Depressive Episode, Persistent Depressive Disorder, Posttraumatic Stress Disorder, Generalized Anxiety Disorder, Alcohol Use Disorder, and Adjustment Disorder. To minimize participant burden and avoid dropouts, only screening questions were used for: Panic Disorder, Agoraphobia, Social Anxiety Disorder, Specific Phobia, Social Phobia, non-alcohol Substance Use Disorder, and Sleep Disorders.

Interviews were conducted by 30 study nurses who had attended a full-day training session led by a senior researcher from the University of Mainz (SS), a licensed psychotherapist with extensive SCID experience. This initial training was followed by monthly review sessions, led by the project coordinators (MH, MS). The SCID demonstrated an inter-rater reliability of $\upkappa =$ 0.8 to 0.95 in previous studies [20, 21].

For the rationale, conduct, and results of the SCID interviews, see Singer et al. [22].

#### The distress thermometer

The DT, developed by the National Comprehensive Cancer Network (NCCN) [11, 12], is a questionnaire for cancer patients to evaluate their distress level on a visual analogue scale from 0 (no distress) to 10 (extreme distress), accompanied by a 36-item problem list. We only used the visual analogue scale here. A cutoff value of ≥ 5 is established to indicate clinically relevant distress [12]. For psychometric properties, see Appendix A2.

#### The emotional-functioning scale of the EORTC QLQ-C30

The EORTC QLQ-C30 [13] is a questionnaire to assess quality of life in cancer patients. We used its EF scale, which comprises the questions: “Did you feel tense? Did you worry? Did you feel irritable? Did you feel depressed?”.

Likert-scale (1–4) responses are summed and transformed into scores from 0 to 100 [19], higher scores indicating better emotional functioning. According to Giesinger eta al. [23], scores < 71 indicate clinically relevant impairments in EF. Previous Studies indicated a Cronbach’s Alpha of 0.85 [24, 25]. For more psychometric properties, see Appendix S2.

#### The three screening questions

The TSQ were developed by Voß et al. [5] to assess the psychosocial and physical health status of glioma patients who might struggle with questionnaires due to neurocognitive deficits. It compiles three yes/no-questions:

1. Has your mood worsened?
2. Do physical changes put strain on you?
3. Has your memory capacity worsened?

Healthcare professionals asked these questions during consultations and recorded responses, with methods varying by clinical need. Some physicians recorded yes/no answers, while others provided text responses, which were later jointly dichotomized by the project coordinators.

Responses can be summed up to a numeric score from 0 to 3, with higher scores indicating higher distress. This score is referred to as the ***TSQ score***.

### Statistical analyses

First, descriptive statistics were reported for the number of patients per group, diagnosed glioma types, and age and sex distributions, followed by information on the presence of mental comorbidities and summary statistics on TSQ, DT and EF scores.

Associations between each score and the psychiatric comorbidity were visualized using stacked bar plots and, to illustrate the direction of association, quantified using point-biserial correlations.

Discriminatory abilities were evaluated by receiver operating characteristic (ROC) curves and corresponding areas under the curves (AUCs). Moreover, maximum Youden indices (calculated as Sensitivity + Specificity – 1) with corresponding threshold were reported. For the DT and EF, Youden indices based on the established thresholds of 5 and 71 [12, 23], respectively, were specified. Using bootstrapping (10,000 iterations; see Appendix A3 for details), 95% confidence intervals (CIs) were calculated for sensitivity–specificity pairs, AUCs, and Youden indices. Finally, results from the screening tools were compared, and bootstrapping was used to test whether AUCs differed significantly between each pair.

To address the fact that screening tools were administered to fully or partially distinct patient groups, we conducted a sensitivity analysis (see Appendix A4) comparing the discriminatory ability of the EF scale with that of the TSQ (in the IG) and the DT (in the CG), respectively, while a more detailed discussion on the implications of disjoint groups is provided in the Appendix (A5).

For missing data, no imputations were performed, and observation pairs in which either the SCID or the screening tool had missing values were excluded from the analysis. For details on missing data and their mechanisms, see Appendix A6.

All analyses were conducted in R, using the pROC package [26] for ROC analyses.

## Results

### Sample characteristics

The study included 763 patients, a total of 354 in the IG and 409 in the CG. In total, 60.9% of all patients were diagnosed with glioblastoma WHO grade IV (Table 1), 24.3% with astrocytoma WHO grade III, and 12.4% with oligodendroglioma WHO grade III. The study was planned and initiated in 2019, with diagnoses documented according to the 2016 WHO classification, using Roman numerals instead of Arabic numbers. However, 18 patients (2.4%) had been diagnosed with an anaplastic oligoastrocytoma under the 2007 WHO classification, and it was not feasible to update these diagnoses to align with the 2016 WHO criteria.

**Table 1:** Summary statistics of all important variables, including baseline characteristics of the patient population, the presence of mental comorbidities and the different screening tools

Item	Overall, *N = *763	IG, *N = *354	CG, *N = *409
Baseline Variables			
Age in years, t1, physician			
Median, Mean (SD)	54, 53.0 (13.7)	53, 51.9 (13.9)	55, 53.9 (13.4)
Range	19—86	19—86	20—84
Sex, t1, physician			
Male	431 (56.5%)	202 (57.1%)	229 (56.0%)
Female	331 (43.4%)	152 (42.9%)	179 (43.8%)
Diverse	1 (0.1%)	0 (0.0%)	1 (0.2%)
Professional qualification, t2, self-report			
Training	285 (43.1%)	124 (37.8%)	161 (48.3%)
Technical/Master school	73 (11.0%)	45 (13.7%)	28 (8.4%)
Technical College	71 (10.7%)	33 (10.1%)	38 (11.4%)
University	160 (24.2%)	88 (26.8%)	72 (21.6%)
Other	37 (5.6%)	21 (6.4%)	16 (4.8%)
None	35 (5.3%)	17 (5.2%)	18 (5.4%)
Missing	102	26	76
Diagnosis, t1, physician			
Oligodendroglioma WHO grade I I	94 (12.4%)	46 (13.0%)	48 (11.9%)
Oligoastrocytoma WHO grade III	18 (2.4%)	8 (2.3%)	10 (2.5%)
Astrocytoma WHO grade III	184 (24.3%)	92 (26.0%)	92 (22.8%)
Glioblastoma WHO grade IV	462 (60.9%)	208 (58.8%)	254 (62.9%)
Missing	5	0	5
Stage of disease, t1, physician			
Primary Tumor	538 (70.6%)	244 (68.9%)	294 (72.1%)
Recurrence	224 (29.4%)	110 (31.1%)	114 (27.9%)
Missing	1	0	1
Time since first diagnosis in months, t1, physician			
Median, Mean (SD)	0, 1.3 (1.7)	0, 1.4 (1.8)	0, 1.1 (1.6)
Range	0—10.2	0—9.4	0—10.2
Missing	13	5	8
Situation (MRI) according to RANO criteria, t1, physician			
Complete response	138 (18.2%)	66 (18.7%)	72 (17.8%)
Partial response	85 (11.2%)	34 (9.6%)	51 (12.6%)
Stable disease	419 (55.4%)	203 (57.5%)	216 (53.5%)
Progressive disease	115 (15.2%)	50 (14.2%)	65 (16.1%)
Missing	6	1	5
Karnofsky-Index, t1, physician			
Median, Mean (SD)	90, 85.2 (12.4)	90, 87.1 (11.9)	90, 83.6 (12.6)
Range	40—100	40—100	40—100
Missing	5	3	2
Study Variables			
Comorbid Psychiatric Disorders (according to the SCID), t2, study nurse			
Median, Mean (SD)	0, 0.36 (0.60)	0, 0.47 (0.62)	0, 0.26 (0.56)
Range	0—4	0—4	0—3
Absent	474 (68.6%)	200 (58.3%)	274 (78.7%)
Present	217 (31.4%)	143 (41.7%)	74 (21.3%)
Missing	72	11	61
Question 1, Has your mood worsened?, t1, physician			
No		135 (38.9%)	
Yes		212 (61.1%)	
Missing		7	
Question 2, Do physical changes put strain on you?, t1, physician			
No		103 (29.3%)	
Yes		248 (70.7%)	
Missing		3	
Question 3, Has your memory capacity worsened?, t1, physician			
No		138 (39.5%)	
Yes		211 (60.5%)	
Missing		5	
Sum of the three Screening Questions (TSQ Score), t1, physician			
Median, Mean (SD)		2, 1.92 (1.0)	
Range		0—3	
0		39 (11.4%)	
1		70 (20.4%)	
2		113 (32.9%)	
3		121 (35.3%)	
Missing		11	
EORTC QLQ-C30, Emotional-Functioning (EF) Scale, t1, self-report			
Median, Mean (SD)	58, 59.2 (28.1)	58, 59.0 (28.4)	58, 59.3 (27.8)
Range	0—100	0—100	0—100
< 71	288 (38.4%)	134 (38.2%)	154 (38.6%)
$\ge$ 71	462 (61.6%)	217 (61.8%)	245 (61.4%)
Missing	13	3	10
NCCN Distress Thermometer (DT), t1, self-report			
Median, Mean (SD)			5, 5.0 (2.6)
Range			0—10
< 5			151 (38.9%)
$\ge$ 5			237 (61.1%)
Missing			21

*SD* Standard Deviation, *WHO* World Health Organization, *MRI* Magnetic Resonance Imaging, *RANO* Response Assessment in Neuro-Oncology, *EORTC* European Organisation for Research and Treatment of Cancer, *QLQ-C30* Quality of Life Questionnaire Core 30, *NCCN* National Comprehensive Cancer Network, t1, t2, and t3 denote the time points at which each variable was collected

Variables labeled *physician*/*study nurse* were recorded by the responsible physician or study nurse, respectively, while variables labeled *self-report* were obtained through self-report questionnaires

Mean age was 53.0 years (SD = 13.7, Range 19–86). The proportion of male patients (56.5%) was higher than that of female patients (43.4%). Further characteristics, including the source (physician, study nurse, or patient self-report) and the time point of collection for each variable, are provided in Table 1.

### Sample representativeness

Our sample reflects national cancer registry demographics in terms of sex and age (57% women vs. 55% nationally; www.krebsdaten.de, accessed August 2, 2024). Approximately 45% of eligible patients declined participation, often due to emotional distress, likely resulting in an underrepresentation of highly distressed individuals. However, this does not bias our main estimate, as the association between screening tools and SCID diagnoses is not dependent on prevalence.

### Comorbid psychiatric disorders

Of the 763 patients enrolled, 702 agreed to be interviewed using the SCID, and 691 (90.6%) were ultimately interviewed. The main reason for missing interviews was COVID-related disruptions (see Appendix A6 for more details). On average, the interviews lasted 17 min.

Of the interviewed patients, 31.4% were diagnosed with at least one comorbid mental disorder. Notably, the proportion of mental comorbidity differed substantially between groups (IG: 41.7%, CG: 21.3%). This imbalance could not be explained by any of the surveyed variables, and the underlying reasons remain unclear.

Exactly one common mental disorder was diagnosed in 27.4% of patients. Diagnoses of multiple comorbidities were rare: 3.2% had two, 0.4% had three, and 0.1% had four. On average, the number of comorbid mental disorders was 0.36 across all patients, and 1.16 among those with at least one diagnosis.

The most frequently diagnosed disorder was PTSD (*n = *68, 9.8%), which was often cancer-related (for details, see Appendix A7), followed by persistent depressive disorder (*n = *57, 8%) and generalized anxiety disorder (*n = *50, 7%). Fewer patients were diagnosed with adjustment disorder (*n = *29, 4%) or alcohol use disorder (*n = *8, 1%).

For details, please refer to Singer et al. [20].

### Presence of increased distress, impaired emotional functioning, and need for support

In total, 61.1%, 70.7%, and 60.5% of patients answered questions 1, 2 and 3 of the TSQ affirmatively (eFIG 1a), with a mean TSQ score of 1.9 (SD = 1.0, Range 0–3, media*n = *2). 304 patients responded affirmatively to at least one of the three questions (88.6%, eFIG 1b). Cronbach’s alpha of the TSQ score was 0.48.

The mean DT score was 5.0 (SD = 2.6, Range 0–10, media*n = *5; eFIG 1c). A total of 61.1% of patients indicated a distress level of ≥ 5.

The mean EF level was 59.2 (SD 28.1, Range 0–100, media*n = *58.3). The proportion of patients with impaired EF (≤ 71) was 61.6% (eFIG 1d).

The Pearson correlation between DT and EF was moderately strong r = – 0.55 (95% CI: –0.62, –0.29; *p < *0.001), and moderate between TSQ scores and EF r = – 0.44 (95% CI: –0.52, –0.35; *p < *0.001).

The numbers of valid responses were 343 (of *n = *354, IG) for the TSQ, 388 (of *n = *409, CG) for the DT and 750 (of *n = *763) for the EF scale.

### Relationship between the presence of psychiatric comorbidity and the TSQ

**Fig. 1:**
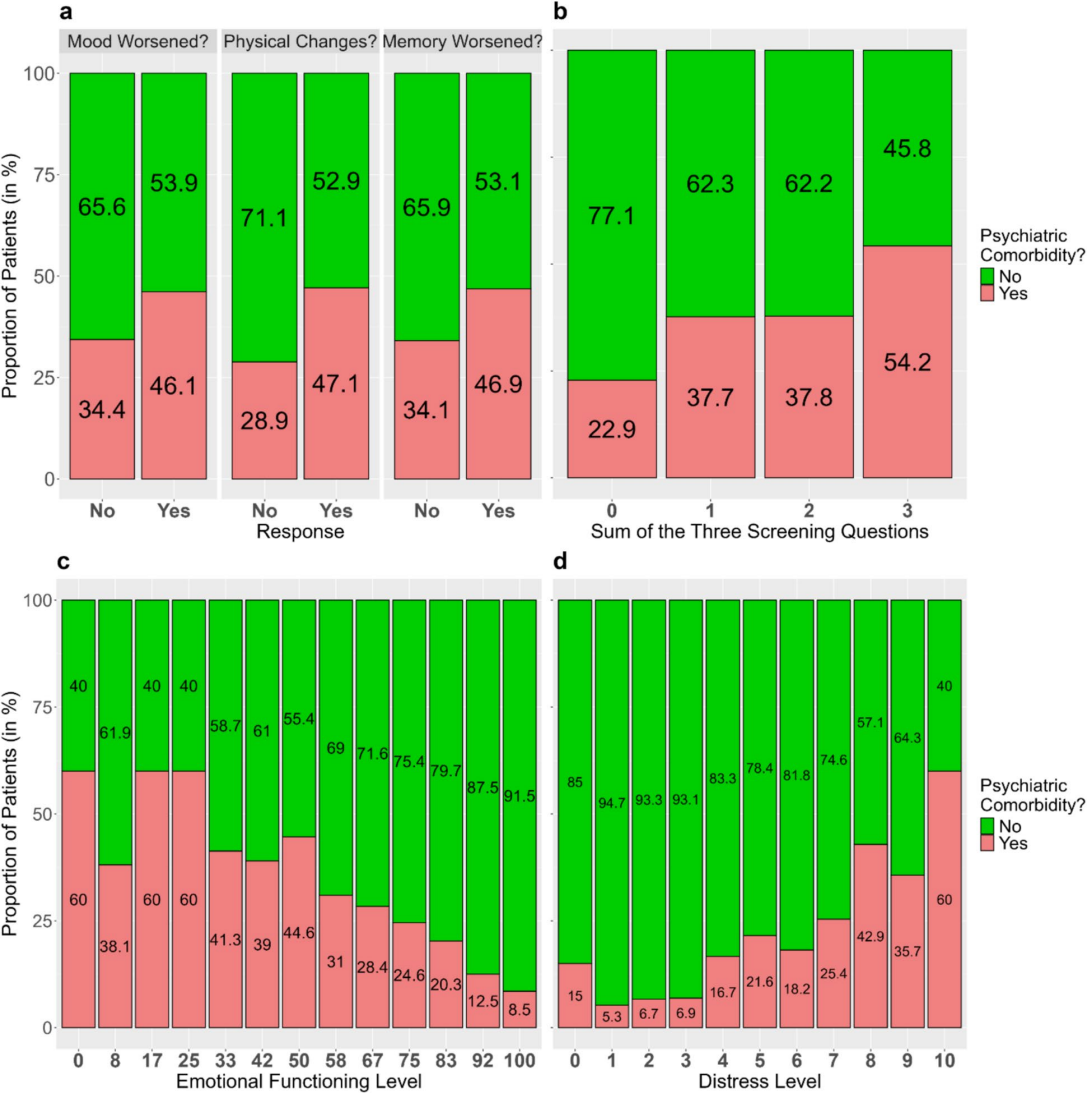
Proportions of patients with psychiatric comorbidity based on their response to each of the three screening questions (TSQ) (**a**) Proportions of patients with psychiatric comorbidity according to their TSQ score (**b**) Proportions of patients with psychiatric comorbidity according to their emotional functioning levels (note that, for the sake of clarity, EF scores with fewer than five observations were omitted) (**c**) Proportions of patients with psychiatric comorbidity according to their distress levels (**d**)

For each of the TSQ, the proportion of patients with comorbidity was higher among patients who answered affirmatively (Fig. 1a). The point-biserial correlations were weak, with values of r = 0.12 (95% CI: 0.01–0.22, p = 0.03), r = 0.17 (95% CI: 0.06–0.27, p = 0.002), and r = 0.13 (95% CI: 0.02–0.23, p = 0.02).

**Fig. 2:**
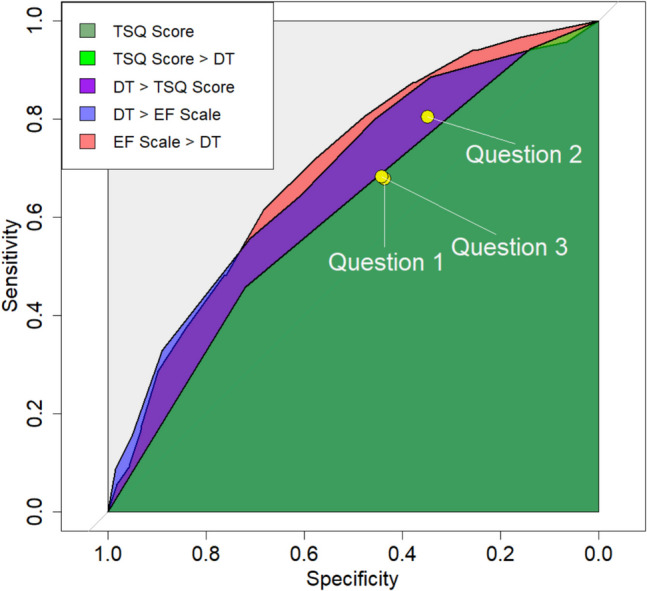
Comparison of receiver operating characteristic (ROC) curves and areas and the curves (AUCs) for all considered screening tools. The three yellow dots represent the sensitivity–specificity pairs obtained from questions 1–3. The combined dark and light green areas depict the AUC of the TSQ (three screening questions) score. The light green area alone indicates the region at which the TSQ score surpasses the DT (distress thermometer). The combined purple and blue areas show the regions at which the DT outperforms the TSQ score. The blue area alone represents the region at which the DT outperforms the EF (emotional functioning) scale, while the red area denotes the region at which the EF scale surpasses the DT

For questions 1–3, the corresponding sensitivity–specificity pairs were 0.68/0.44, 0.80/0.35, and 0.68/0.44, respectively (Fig. 2, for a black-and-white version see eFIG 5). AUCs were 0.56, 0.58, and 0.56 (95% CI: 0.51–0.61, 95% CI: 0.53–0.62, 95% CI: 0.51–0.61; Table 2).

**Table 2:** The areas under the receiver operating characteristic (ROC) curves (AUCs) for each instrument, along with their corresponding 95% confidence intervals (CIs) and the p-values obtained from bootstrapping to test the hypothesis that the two true AUCs are equal

	Q1. Mood	Q2. Physical	Q3. Memory	TSQ Score	DT	EF scale
AUC	0.56	0.58	0.56	0.61	0.69	0.70
AUC, 95% CI	0.51–0.61	0.53–0.62	0.51–0.61	0.55–0.66	0.62–0.76	0.66–0.74
Q1. Mood		p $\approx$ 0.59	p $\approx$ 0.90	p $\approx$ 0.22	p $\approx$ 0.004	*p < *0.001
Q2. Physical			p $\approx$ 0.54	p $\approx$ 0.58	p $\approx$ 0.001	*p < *0.001
Q3. Memory				p $\approx$ 0.12	p $\approx$ 0.005	*p < *0.001
TSQ Score					p $\approx$ 0.09	p $\approx$ 0.009
DT						p $\approx$ 0.75

Abbreviaons: *Cor*. Correlation, Q1. Mood = Question (1) “Has your mood worsened?”; Q2. Physical = Question (2) “Do physical changes put strain on you?”; Q3. Memory = Question (3) “Has your memory capacity worsened?”; *TSQ* Three Screening Questions, *DT* distress thermometer, *EF* emotional functioning scale

The TSQ score was weakly positively associated with psychiatric comorbidity (r = 0.19, 95% CI: 0.08–0.29, *p < *0.001; Fig. 1b). The AUC of the ROC curve was 0.61 (95% CI: 0.55–0.66; Table 2, eFIG 2). The maximum Youden index of 0.18 (95% CI: 0.09–0.28), corresponding to a sensitivity/specificity of 0.46/0.72, was observed at a threshold of ≥ 3.

All sensitivity–specificity pairs and their 95% CIs can be found in the Appendix (eTable 1).

### Relationship between the presence of psychiatric comorbidity and the DT

The level of distress was relatively weakly positively associated with the presence of psychiatric comorbidity (r = 0.26, 95% CI: 0.16–0.36, *p < *0.001). About 5–7% of patients with distress levels up to $\le$ 3 were diagnosed with a psychiatric disorder, in contrast to 40–60% of those with a level of ≥ 8 (Fig. 1b). Yet, a substantial proportion of patients did not fit this pattern. For instance, more than 40% of patients who reported the maximum distress level of 10 were not diagnosed with any mental comorbidities, while a notable 15% of patients who reported no distress at all were diagnosed with a mental disorder.

The AUC of the ROC curve was 0.69 (95% CI: 0.62–0.76; Table 2, eFIG 3). The threshold of ≥ 5 gave a Youden index of 0.26 (95% CI: 0.14–0.36), with a sensitivity/specificity 0.80/0.46. The maximum Youden index of 0.27 (95% CI: 0.19–0.40) was observed at a threshold of ≥ 7, with a corresponding sensitivity/specificity of 0.56/0.71.

A table with all sensitivity–specificity pairs and their 95% CIs using can be found in Appendix (eTable 2).

### Relationship between the presence of psychiatric comorbidity and the EF scale

EF levels and the presence of psychiatric comorbidity were weakly to moderately negatively associated with r = – 0.32 (95% CI: – 0.38, – 0.25, *p < *0.001). While more than half of the patients with EF levels between 0 and 25 had a common mental disorder, they were rare among patients with an EF level of 100 (8.5%; Fig. 1d). At the same time, however, 40% of patients with the lowest level of emotional functioning were not diagnosed with a mental comorbidity, while 8.5% of those reporting the highest level of emotional functioning were.

The AUC was 0.70 (95% CI: 0.66—0.74; Table 2, eFIG 4). The threshold of 71 resulted in a Youden index of 0.28 (95% CI: 0.21–0.35), obtained with a sensitivity/specificity of 0.81/0.46. The maximum Youden index of 0.30 (95% CI: 0.24–0.38) was achieved at thresholds of 52.8 and 56.9, with corresponding sensitivity/specificity of 0.62/0.68.

A table with all sensitivity–specificity pairs and their 95% CIs using can be found in Appendix (eTable 3).

### Comparison of discriminatory performances

The sample ROC curve of the EF scale fully encompassed, and that of the DT almost fully encompassed, those of questions 1–3 and the TSQ score (Fig. 2).

Comparing the DT and EF scale, the EF performed better in high-sensitivity regions, while the DT showed slightly superior discriminatory ability in high-specificity regions; however, differences in those regions were negligible.

Using bootstrapping, (Table 2), the true AUC based on the EF scale was significantly higher than those based on questions 1–3 and the TSQ score (*p < *0.001, *p < *0.001, *p < *0.001, p ≈ 0.009). These p-values, as well as all subsequent ones, refer to pairwise comparisons among the screening tools. Similarly, there was strong to moderate evidence that the true AUC of the DT was higher than those of questions 1–3 and the TSQ score (p ≈ 0.004, 0.001, 0.005, 0.09). Yet AUCs based on the EF scale and the DT did not differ significantly (p ≈ 0.75). Furthermore, although the TSQ score had a higher AUC than questions 1–3, the differences were not statistically significant (p ≈ 0.22, 0.58, 0.12).

Sensitivity analyses within the IG and CG subgroups showed similar results (Appendix A4), confirming the main findings. Notably, the EF scale's AUC was moderately higher in the CG than in the IG (0.73 vs. 0.69).

## Discussion

This study compared the TSQ, DT, and EF scale in identifying SCID-diagnosed mental comorbidities in patients with high-grade glioma. Although elevated levels of distress often occur without the presence of a mental health diagnosis, thus necessitating psychosocial support, reimbursement for these services, at least by psychotherapists in private practice, is only provided by the health insurances if such a diagnosis is given [8, 9].

Based on AUC and Youden index, the EF scale performed slightly—but not significantly—better than the DT; both outperformed the TSQ score and questions 1–3. However, despite exceeding chance levels, none demonstrated strong discriminatory power. It should be kept in mind that, unlike the self-report DT and EF scale, the TSQ are clinician-administered and partly judgment-based, limiting its direct comparability.

A secondary goal of this study was to determine whether the TSQ was more effective as a composite score or individual items. Although the composite score showed better discrimination than questions 1–3, its low internal consistency (α = 0.48) suggests the items may be more useful on their own.

Compared to our results, other studies using the SCID as the reference standard have reported AUCs substantially higher than those observed in our study. Based on the DT, Ryan et al. [15] (2011) and Recklitis et al. [16] (2016) likewise reported AUCs around 0.80, notably higher than the 0.69 observed in our study. Using the EF scale, Singer et al. [17] reported an AUC of approximately 0.80, in contrast to the 0.70 observed in this study.

Differences in performance may reflect variation in study populations, levels of emotional distress, and the SCID modules used. Regarding the Distress Thermometer (DT), Ryan et al. surveyed 205 patients with advanced cancer using SCID modules on mood, anxiety, adjustment, and optional disorders, reporting a psychiatric comorbidity rate of 12.7% and a mean distress level of 4.01. Recklitis et al. assessed 247 young cancer survivors using the research version of the SCID, including the same modules as Ryan et al., with the addition of trauma-related, obsessive–compulsive, and minor depressive disorders. They reported a higher comorbidity rate (17.8%) but lower distress (3.04). Regarding the Emotional Functioning (EF) scale, Singer et al. surveyed 250 patients with laryngeal cancer, reporting a comparable comorbidity rate of 19.8% and a mean score of 5.2 on the mood version of the Visual Analogue Scale (VAS) [27].

In contrast, our study showed a higher prevalence of psychiatric disorders (31.4%) and distress levels (4.97), consistent with the emotional burden of glioma. [28–30]. Meta-analyses indicate that approximately 30% of cancer patients experience mental health conditions, aligning with our findings.

The DT or EF scale could guide initial screening in glioma patients, with SCID interviews limited to those above certain thresholds. However, while some thresholds (e.g., EF: 94% sensitivity, 25% specificity) meet sensitivity requirements, they reduce SCID use only marginally and risk many false negatives. This approach is mainly suitable when resources are limited, in which case Youden-maximizing or established cutoffs (DT: 5; EF: 71) may be most practical.

The screening tools'limited discriminative power likely reflects the conceptual distinction between distress/functioning and mental disorders. One can meet criteria for a diagnosis yet feel emotionally well, or struggle emotionally without a diagnosis [31, 32]— a pattern also evident in our data.

### Limitations

Our results should be interpreted in the light of the study’s limitations.

First, the DT and the TSQ were evaluated in different patient groups, making comparisons challenging and partially necessitating unpaired tests with generally lower statistical power.

Additionally, a significant difference was observed in the prevalence of psychiatric comorbidity between the groups. The reasons for this discrepancy remain unknown. Since positive and negative predictive values depend on prevalence, these were not reported. While associations, and sensitivity/specificity remain unaffected, similar group distributions might have reduced estimation uncertainty.

A more detailed discussion of these limitations can be found in the Appendix (A5).

Moreover, SCID interviews were carried out by study nurses rather than by mental health professionals. Despite thorough training and supervision, some degree of misclassification in either direction cannot be ruled out.

Furthermore, for some SCID modules (for details, see the Method section), only the initial screening items were administered. This approach aimed to reduce participant burden, as longer interviews might have discouraged participation in this vulnerable patient group. Consequently, because the full SCID was not conducted, the true prevalence of mental disorders in the sample may have been underestimated.

Lastly, as participation required consent, our sample may not fully represent all glioma patients.

## Conclusion

This study examined the potential of three different screening tools to predict the presence of at least one psychiatric disorder in patients with high-grade glioma.

The EF scale showed the strongest discrimination, followed by the DT, while the TSQ and its score performed notably worse. Despite this, the EF and DT lacked sufficient accuracy to reliably predict SCID-assessed mental comorbidity in glioma patients. Their use may be justified only when SCID interviews are impractical or must be significantly shortened, despite the increased risk of false negatives.

## Supplementary Information

Below is the link to the electronic supplementary material.

Supplementary file1 (DOCX 287 KB)

## Data Availability

Research data are stored in an institutional repository and will be shared upon reasonable request to the first and last authors.
