# Supplementary material for: Distress screening in patients with high-grade glioma: diagnostic accuracy in relation to a structured clinical interview in a multicenter cluster-randomized controlled trial
Source: Support Care Cancer. 2025 Jul 31;33(8):737. doi: 10.1007/s00520-025-09810-1 (PMC12313720; doi:10.1007/s00520-025-09810-1)
Supplement: Supplementary file 1 — Supplementary file1 (DOCX 287 KB) [file 520_2025_9810_MOESM1_ESM.docx]

**Supplementary Information**


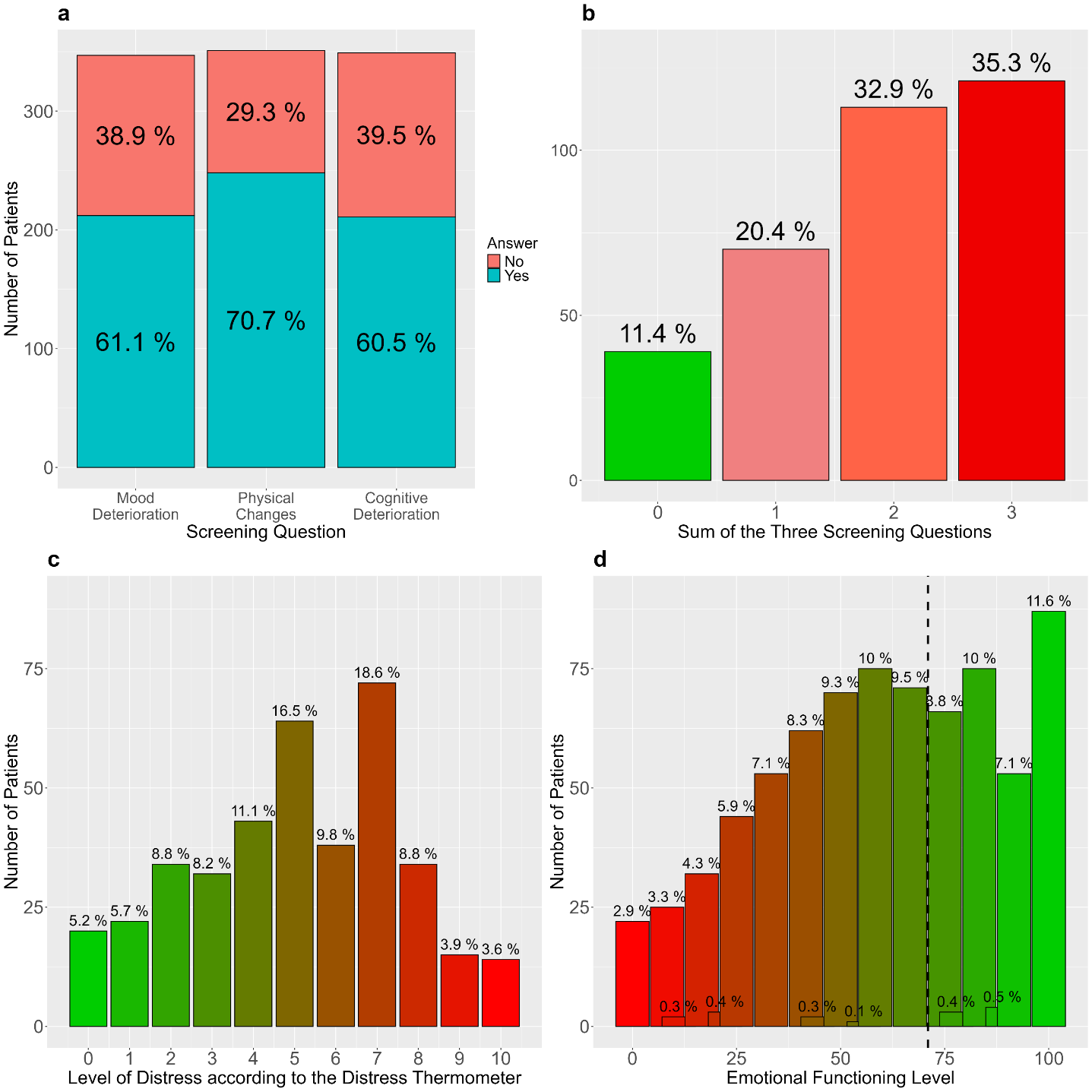


***eFIG 1*** *Distribution of the observed answers to each of the three screening questions (TSQ) (****a****). Distribution of the observed TSQ scores (****b****). Distribution of the observed distress levels according to the distress thermometer (****c****). Distribution of the observed emotional-functioning level (****d****).*


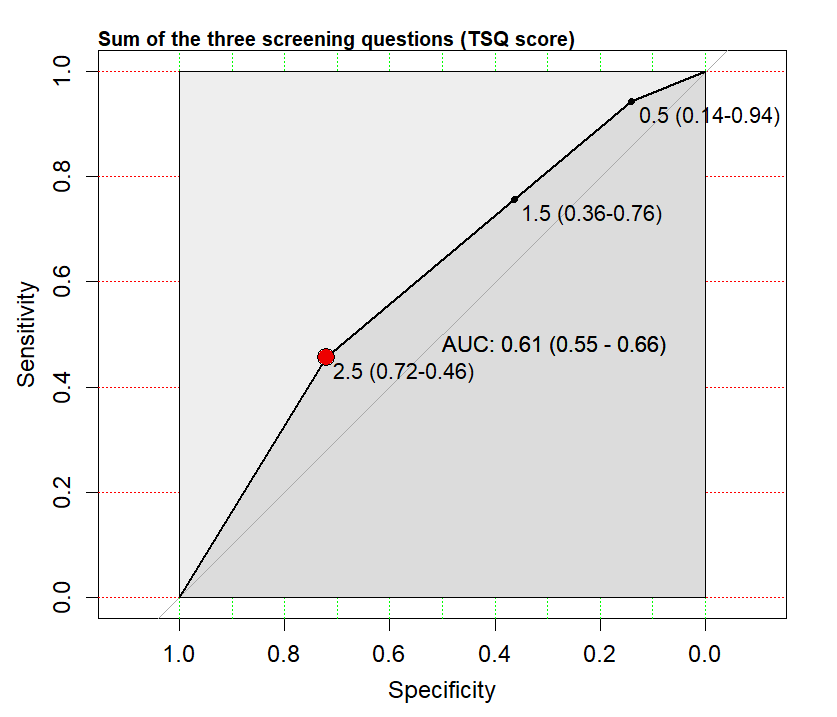


***eFIG 2*** *Receiver operating characteristic (ROC) curve obtained based on the three-screening-question (TSQ) score with the corresponding area under the curve (AUC) and its 95% confidence interval. Also given is the cutoff score that maximizes the Youden index and its corresponding sensitivity-specificity pair (ret dot). Note that, for example, here >0.5 is equivalent to ≥1*


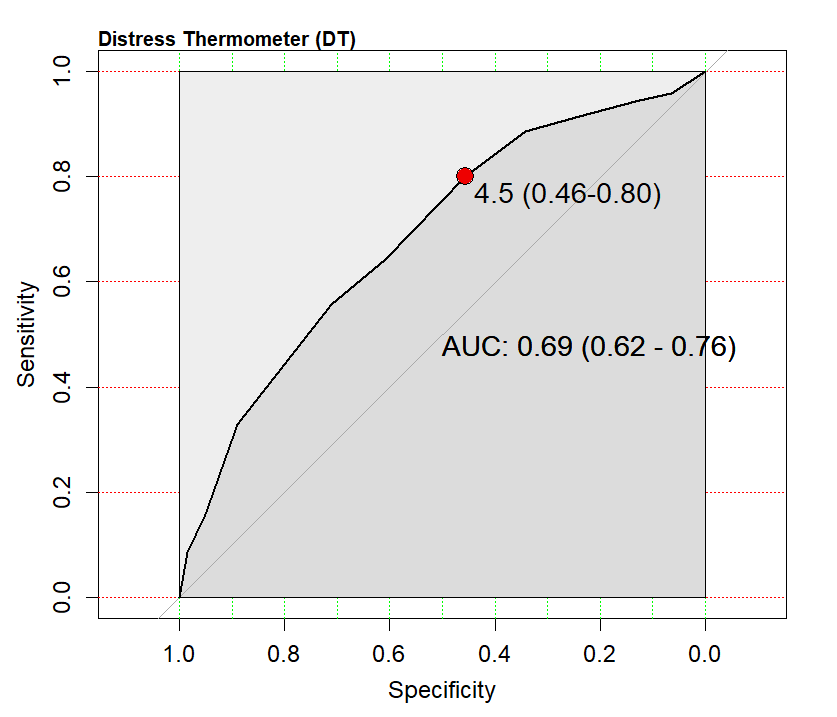


***eFIG 3*** *Receiver operating characteristic (ROC) curve obtained based on the distress thermometer (DT) with the corresponding area under the curve (AUC) and its 95% confidence interval. Also given is the well-established cutoff score of 5 with its corresponding sensitivity-specificity pair (ret dot). Note that, for example, here >4.5 is equivalent to ≥5*


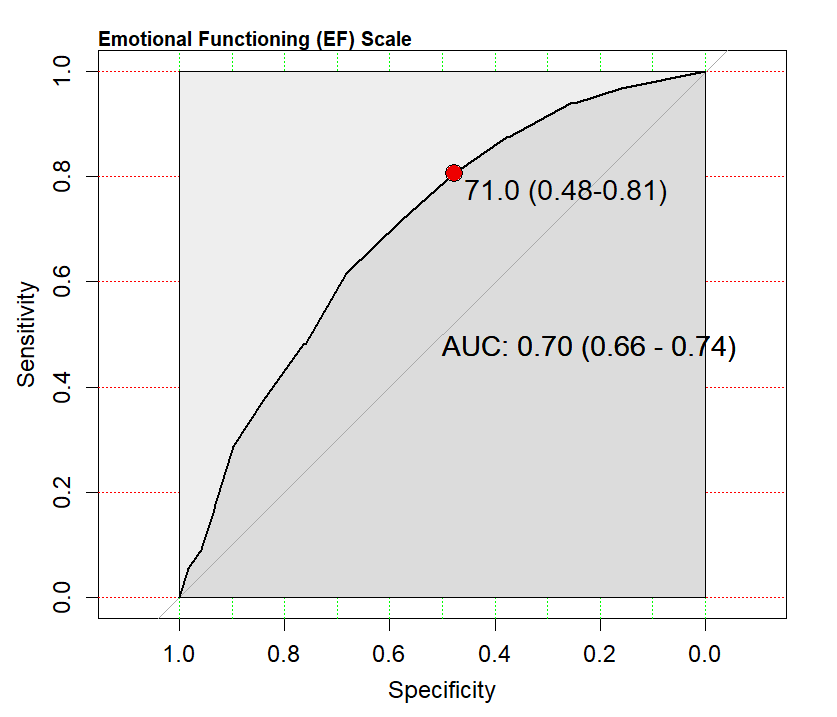


***eFIG 4*** *Receiver operating characteristic (ROC) curve obtained based on the emotional functioning (EF) scale with the corresponding area under the curve (AUC) and its 95% confidence interval. Also given is the well-established cutoff score of 71 with its corresponding sensitivity-specificity pair (ret dot)*


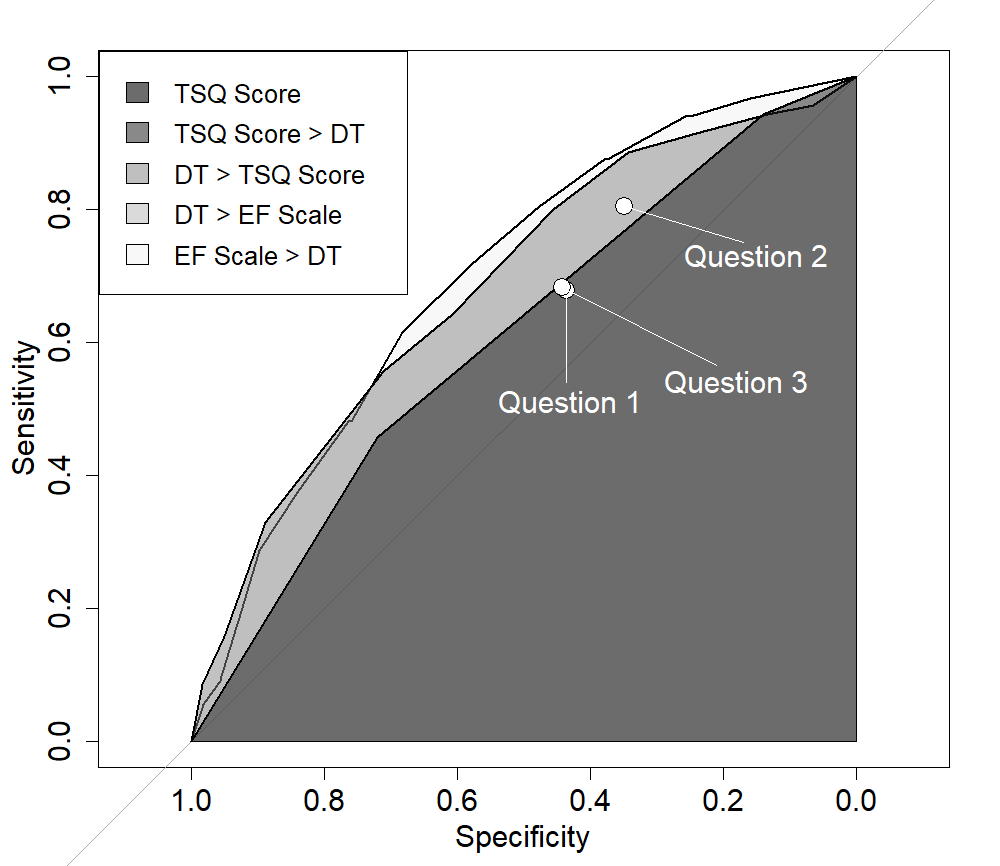


**eFIG 5** Comparison of receiver operating characteristic (ROC) curves and areas under the curves (AUCs) for all considered screening tools. The three white dots represent the sensitivity–specificity pairs obtained from questions 1–3. The dark grey area depicts the AUC of the TSQ (three screening questions) score. The slightly lighter grey area indicates the region where the TSQ score outperforms the DT (Distress Thermometer). The moderately light grey area shows where the DT outperforms the TSQ score. The area filled with an even lighter grey hue represents the region where the DT outperforms the EF (Emotional Functioning) scale, while the lightest grey area indicates where the EF scale surpasses the DT

***eTable 1*** *Pairs of sensitivity and specificity that are obtained when all patients whose distress level - according to the distress thermometer (DT) - exceeds the listed cutoff score are classified as having a mental disorder according to the Structured Clinical Interview for DSM (Diagnostic and Statistical Manual of Mental Disorders, DSM) (SCID)*

| TSQ score | ≥ 1 | ≥ 2 | ≥ 3 |
| --- | --- | --- | --- |
|  | | | |
| 95 % CI, Upper | 97.9 | 82.9 | 54.3 |
| Sensitivity (%) | 94.3 | 75.7 | 45.7 |
| 95 % CI, Lower | 90.0 | 68.6 | 37.9 |
|  | | | |
| 95 % CI, Upper | 19.2 | 43.0 | 78.2 |
| Specificity (%) (%) | 14.0 | 36.3 | 72.0 |
| 95 % CI, Lower | 9.3 | 29.5 | 65.3 |

***eTable 2*** *Pairs of sensitivity and specificity that are obtained when all patients whose distress level - according to the distress thermometer (DT) - exceeds the listed cutoff score are classified as having a mental disorder according to the Structured Clinical Interview for DSM (Diagnostic and Statistical Manual of Mental Disorders, DSM) (SCID)*

| Distress Level | ≥ 1 | ≥ 2 | ≥ 3 | ≥ 4 | ≥ 5 | ≥ 6 | ≥ 7 | ≥ 8 | ≥ 9 | ≥ 10 |
| --- | --- | --- | --- | --- | --- | --- | --- | --- | --- | --- |
|  | | | | | | | | | | |
| 95 % CI, Upper | 100 | 98.6 | 97.1 | 95.7 | 88.6 | 75.7 | 67.1 | 44.3 | 24.3 | 15.7 |
| Sensitivity (%) | 95.7 | 94.3 | 91.4 | 88.6 | 80.0 | 64.3 | 55.7 | 32.9 | 15.7 | 8.6 |
| 95 % CI, Lower | 90.0 | 88.6 | 84.3 | 80.0 | 70.0 | 52.9 | 44.3 | 22.9 | 7.1 | 2.9 |
|  | | | | | | | | | | |
| 95 % CI, Upper | 9.5 | 17.5 | 29.3 | 39.9 | 51.7 | 66.5 | 76.4 | 92.8 | 97.3 | 99.6 |
| Specificity (%) (%) | 6.5 | 13.3 | 24.0 | 34.2 | 45.6 | 60.8 | 71.1 | 89.0 | 95.1 | 98.5 |
| 95 % CI, Lower | 3.8 | 9.5 | 19.0 | 28.5 | 39.5 | 55.1 | 65.4 | 85.2 | 92.4 | 97.0 |

***eTable 3****: Pairs of sensitivity and specificity that are obtained when all patients whose emotional functioning (EF) level falls below the listed cutoff score are classified as having a mental disorder according to the Structured Clinical Interview for DSM (Diagnostic and Statistical Manual of Mental Disorders, DSM) (SCID). Note that, in line with the clinically significant threshold, the cutoff values for this scale—unlike those for the other screening tools—correspond to the mean of the two closest obtainable scores.*

| EF Level | < 4.2 | < 9.7 | < 13.9 | < 19.4 | < 23.6 | < 29.2 | < 37.5 | < 43.1 | < 47.2 |
| --- | --- | --- | --- | --- | --- | --- | --- | --- | --- |
| 95 % CI, Upper | 8.8 | 13.4 | 13.9 | 21.8 | 22.7 | 34.7 | 44 | 54.6 | 54.6 |
| Sensitivity (%) | 5.6 | 9.3 | 9.7 | 16.7 | 17.6 | 28.7 | 37.5 | 48.1 | 48.1 |
| 95 % CI, Lower | 2.8 | 5.6 | 6.0 | 12.0 | 12.5 | 22.7 | 31.0 | 41.2 | 41.2 |
|  | | | | | | | | | |
| 95 % CI, Upper | 99.4 | 97.2 | 97.2 | 95.5 | 95.3 | 92.3 | 87.2 | 80.2 | 79.8 |
| Specificity (%) | 98.3 | 95.5 | 95.5 | 93.4 | 93.2 | 89.8 | 84.0 | 76.4 | 76.0 |
| 95 % CI, Lower | 97.0 | 93.6 | 93.6 | 91.1 | 90.9 | 86.8 | 80.6 | 72.3 | 71.9 |

| EF Level | < 52.8 | < 56.9 | < 62.5 | < 70.8 | < 76.4 | < 80.6 | < 86.1 | < 90.3 | < 95.8 |
| --- | --- | --- | --- | --- | --- | --- | --- | --- | --- |
| 95 % CI, Upper | 68.1 | 68.1 | 77.8 | 85.6 | 91.7 | 91.7 | 96.8 | 96.8 | 99.1 |
| Sensitivity (%) | 61.6 | 61.6 | 71.8 | 80.6 | 87.5 | 87.5 | 94.0 | 94.0 | 96.8 |
| 95 % CI, Lower | 54.6 | 54.6 | 65.7 | 75.0 | 82.9 | 82.9 | 90.7 | 90.7 | 94.0 |
|  | | | | | | | | | |
| 95 % CI, Upper | 72.3 | 72.3 | 62.3 | 52.1 | 42.3 | 41.9 | 29.8 | 28.9 | 19.4 |
| Specificity (%) | 68.3 | 68.3 | 57.9 | 47.7 | 37.9 | 37.4 | 25.7 | 24.9 | 16.0 |
| 95 % CI, Lower | 64.0 | 64.0 | 53.4 | 43.0 | 33.4 | 33.0 | 21.7 | 20.9 | 12.8 |

### Patient Enrolment and Inclusion Criteria

Data collection took place from September 27, 2019, to April 14, 2023. In all 13 participating clinics, patients were enrolled consecutively during their routine appointments. Eligibility criteria included being at least 18 years old, having legal capacity, the ability to give informed consent, and a histologically verified diagnosis of high-grade glioma. Individuals who declined to provide written informed consent after receiving both oral and written information about the study were not included.

During the study period, 1568 patients with glioma attended the clinics and were screened. Of them, 1387 were eligible, 763 participated at t1, 702 agreed to be interviewed, and 691 (50% of all eligible; 91% of all participants) could be interviewed with the SCID. For details see Singer et al. [22].

### Psychometric Properties of the DT and the EF Scale

| Screening Tool | Reference Standard | Cut-off Value | Sensitivity (%) | Specificity (%) | AUC (95% CI) |
| --- | --- | --- | --- | --- | --- |
| DT [12] | HADS ≥ 8 | ≥5 | 77.8% | 47.1% | 0.68 (0.63–0.70) |
| DT [12] | HADS ≥ 11 | ≥5 | 84.5% | 40.7% | 0.72 (0.68–0.77) |
| EF Scale [23] | ABC | <71 | 80.0% | 76.0% | 0.87 (0.84–0.91) |

### Bootstrapping

Bootstrapping, a resampling technique that estimates the sampling distribution of a statistic by repeatedly sampling with replacement from the original dataset, was used extensively in this study.

First, it was employed to compute 95% confidence intervals (CIs) for sensitivity–specificity pairs. Unlike deterministic methods such as Wilson intervals, which treat sensitivity and specificity independently, bootstrapping accounts for their shared data source and thus preserves their correlation—making it more appropriate in this context.

Second, bootstrapping was used to estimate 95% CIs for the maximum Youden indices. For each bootstrap sample, the threshold yielding the highest Youden index was identified. Variability in sample composition led to variation in optimal thresholds and index values, allowing us to assess both the consistency and uncertainty of peak tool performance.

Lastly, bootstrapping was applied to compare the AUCs of screening tools evaluated on different patient groups. Since the DeLong test [33] assumes paired data, it was not applicable. In such unpaired settings, bootstrapping is the only suitable method for testing whether AUCs differ significantly.

### Sensitivity Analysis

As a sensitivity analysis, we conducted DeLong tests to compare AUCs pairwise, including only patients with valid responses to both tools being compared.

We first analyzed the IG subgroup. The EF scale's AUC was 0.69 (95% CI: 0.63–0.74, Table 3), slightly lower than the full-sample AUC (0.70). The DeLong test provided weaker evidence of the EF scale outperforming questions 1–3 or the TSQ compared to bootstrapping. Due to the lower EF scale AUC in this subgroup, the AUCs for questions 1–3 and the TSQ would likely have increased if applied to all patients.

***Table 3*** *Sensitivity analysis results comparing the EF scale with the TSQ (IG) and the DT (CG). Values indicate AUCs; p-values reflect comparisons between the EF scale and the respective screening tool.*

| Intervention Group | |  | Control Group | |
| --- | --- | --- | --- | --- |
| EF scale: 0.69 (95% CI, 0.63 – 0.74) | |  | EF scale: 0.73 (95% CI, 0.67 – 0.79) | |
| Q1. Mood A: 0.56 | p$\approx$0.03 |  | DT: 0.69 | p$\approx$0.34 |
| Q2. Physical: 0.58 | p < 0.001 |  |  |  |
| Q3. Memory: 0.56 | p < 0.001 |  |  |  |
| TSQ Score: 0.61 | p$\approx$0.02 |  |  |  |

Next, we analyzed the CG subgroup. The EF scale yielded an AUC of 0.73 (95% CI: 0.67–0.79, Table 3), slightly higher than in the full sample and notably higher than in the IG. The DeLong test comparing the EF scale and the DT resulted in a p-value of approximately 0.34—lower than the 0.75 from bootstrapping, but still not statistically significant.

Thus, the EF scale demonstrated marginally better discrimination in the CG and slightly worse in the IG compared to the full sample.

### Screening Tool Evaluation Across Uneven Samples

The sensitivity analysis showed that the EF scale’s superior discriminative performance was not merely due to a larger sample size; it remained superior when applied separately to the IG and CG subgroups. While the prevalence of mental comorbidities differed notably between groups (IG: 41.7%, CG: 21.3%), this is not a major concern, as sensitivity and specificity are not influenced by prevalence, and the CG still included 74 cases—enough for robust estimates. Although low outcome prevalence can increase variability and risk of overfitting, the latter is not an issue here, as univariate models are not prone to overfitting.

### Missing Data

Missing data were low for questions 1–3 (2.0%, 0.8%, and 1.4%) and for the TSQ score (3.1%). The EF scale had 1.7% missing data overall, primarily in the CG (2.4%) compared to the IG (0.8%). Missingness was higher for the DT (5.1%) and highest for SCID-based mental comorbidity (9.4%), with rates substantially greater in the CG (14.9%) than in the IG (3.1%). Most missing SCID data (85%) resulted from dropouts after t1, largely due to COVID-related delays and disruptions in some clinics.

Patients with higher distress, lower emotional functioning, or affirmative responses to TSQ question 2 were slightly more likely to have missing EF or SCID data. Since the missingness was related to other variables, it could not be considered completely at random, suggesting a mix of missing at random (MAR) and possibly not missing at random (NMAR) mechanisms.

To address this, multiple imputation was performed, yielding results nearly identical to those from listwise deletion—likely due to the low overall proportion of missing data (<10%). For clarity and simplicity, only the listwise deletion results were presented.

### Prevalence and Origins of PTSD

The elevated prevalence of PTSD in our sample may reflect the traumatic impact of a high-grade glioma diagnosis and its treatment. Among the 68 patients diagnosed with PTSD, 74% reported experiencing their cancer as traumatic, and 59% of those who had multiple traumatic experiences identified their cancer as the most distressing. These findings suggest that cancer-related experiences—such as receiving a terminal prognosis, undergoing invasive brain surgery, or facing neurological decline—can meet SCID trauma criteria.
